# Synovial Chondrosarcoma Arising in Synovial Chondromatosis

**DOI:** 10.1155/2014/647939

**Published:** 2014-03-05

**Authors:** Scott Evans, Michele Boffano, Samena Chaudhry, Lee Jeys, Robert Grimer

**Affiliations:** Royal Orthopaedic Hospital, Birmingham B31 2AP, UK

## Abstract

Primary synovial chondromatosis (SC) is a rare proliferative disorder that causes pain, swelling, and restriction of movement to the joints it affects. The disease frequently runs a protracted course, often requiring multiple surgical procedures to obtain some control. Few reports exist detailing the natural history of SC, although malignant transformation to synovial chondrosarcoma (CHS) is recognized to be a rare event. The aim of our study was to review a large orthopaedic oncology database in order to evaluate the incidence of CHS arising from SC. We identified 78 patients who have presented to our centre with primary synovial chondromatosis (SC). Of those patients, 5 went on to develop malignant change. This represents a 6.4% incidence of developing synovial chondrosarcoma (CHS) within preexisting primary synovial chondromatosis. The patients had a mean age of 28 years at first diagnosis with synovial chondromatosis with the median time from original diagnosis to malignant transformation being 20 years (range 2.7–39 yrs).

## 1. Introduction

Synovial chondromatosis is a rare proliferative, metaplastic disorder of the synovium [1]. The disease was first described by Leannac in 1813 [2]; however, its current description was not applied until 1958 by Jaffe [3]. The exact prevalence of primary synovial chondromatosis (SC) is unknown but it usually affects the third to fifth decades of life [4], with men being affected two to four times more frequently than women [5]. The knee is the most commonly affected large joint followed by hip, shoulder, elbow, ankle, and wrist [6]. Patient's clinical symptoms typically include pain, swelling, and restricted joint movement [7]. The symptoms are often insidious at disease onset and are gradually progressive, although rare spontaneous regression has been reported [8]. Patients often have a protracted length of symptoms prior to diagnosis, with an average of 5 years [9, 10].

Three phases of SC have been described [11]; phase 1 active intrasynovial disease with nodules but no calcifications/intraarticular bodies; phase 2 synovitis with osteochondral nodules in the synovial membrane and loose bodies within the joint; phase 3 multiple loose bodies remain but synovitis is quiescent. This highlights the importance of correlating histological and radiological findings to correctly diagnose the benign entity.

Malignant transformation of primary synovial chondromatosis to synovial chondrosarcoma is recognized to be a rare event with reports estimating the incidence to be in the range of 1–5% [12–14].

The aim of our study was to use a large database of patients with orthopaedic oncology to evaluate the incidence of chondrosarcoma arising from primary synovial chondrosarcoma and to review the time taken for the benign entity to transform into its malignant counterpart.

## 2. Materials and Methods

We conducted a retrospective search of a prospective tumour database to identify all patients treated at our unit with the diagnosis of primary synovial chondromatosis (SC). The diagnosis was made under the auspices of a supraregional multidisciplinary bone tumour unit. Patient demographics were recorded along with the site of primary disease. From this initial search, details of those patients who subsequently developed synovial chondrosarcoma (CHS) were collected.

## 3. Results

A search of our database, which holds prospectively gathered data on over 30,000 patients including over 3800 primary bone sarcomas, identified 78 patients with SC. All had been diagnosed following analysis of their radiological and histological findings between 1980 and 2011. There were 33 females (42.3%) and 45 males (57.7%) with a mean age at presentation with SC of 28 years. The primary site of SC was the knee in 30 patients, hip in 22, hand in 7, shoulder in 6, elbow in 5, foot in 5, wrist in 2, and the ankle in 1.

The median time from original diagnosis with SC to malignant transformation was 20 years (range 2.5–39 years). All patients had undergone multiple procedures in an attempt to control their SC prior to the diagnosis of malignancy. All cases of CHS were diagnosed after careful discussion at a multidisciplinary team meeting involving orthopaedic oncologist, histopathologist, and radiologist (Table 1).

**Figure 1:**
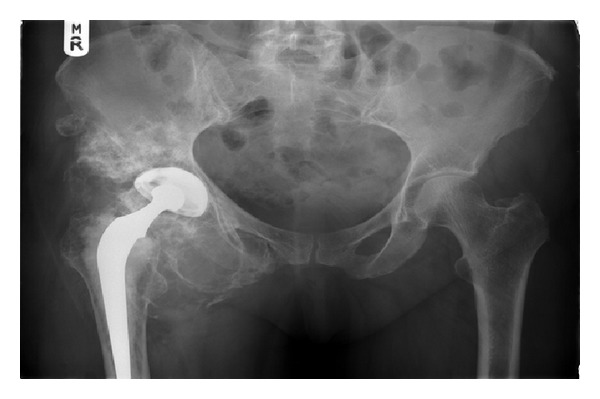
Plain X-ray of case 4 preoperatively.

**Figure 2:**
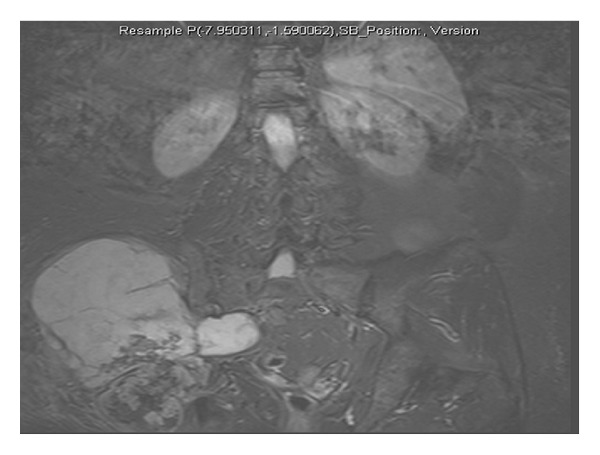
STIR MRI of case 4 preoperatively.

One of the main problems in diagnosing malignancy in SC is that biopsies are often not helpful and clinical features often suggest malignancy more than histology. In two patients (case 1 + 4), repeated biopsy demonstrated benign disease, whilst their imaging studies revealed likely extensive primary synovial chondromatosis. However, it was clear from the patients' level of nonmechanical pain in combination with the rapid and repeated progression of their tumours after debulking that they had aggressive disease. The patients were included in a pragmatic discussion and the decision was made to perform hind-quarter amputation. In both cases, the actual diagnosis of malignancy was only confirmed when the whole tumour had been sampled (see Figures 1 and 2).

Two cases (patients 2 and 3) had large tumours with lung metastases by the time of diagnosis and therefore were treated symptomatically. Both patients subsequently died with chest metastases 3 and 4 years, respectively, after diagnosis. Our one patient with CHS affecting the knee (case 5) underwent an above knee amputation as limb salvage was not possible. Histology revealed a grade II synovial chondrosarcoma. He developed lung metastases 34 months postoperatively and subsequently died 4 years after the amputation.

**Figure 3:**
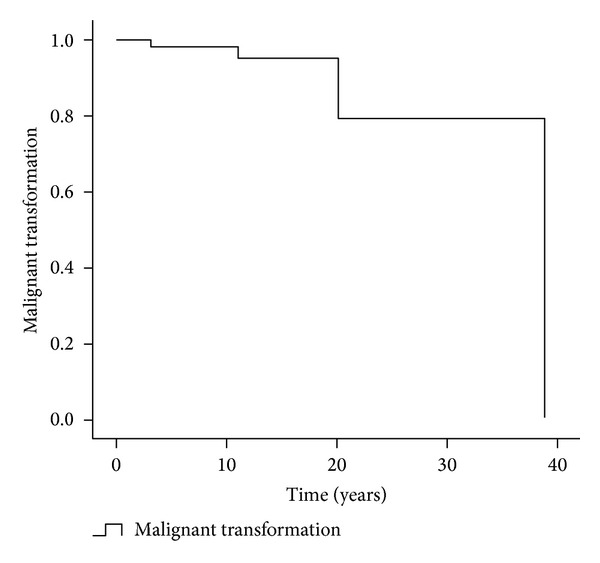
Kaplan-Meier survival (malignant transformation = end-point).

As with other chondrsarcomas, radio/chemotherapy are not typically effective treatments and, therefore, our cases did not receive adjuvant therapy. In summary, 78 patients were identified with SC, with 5 developing CHS. This represents transformation to malignancy in 6.4% of cases. Furthermore, it is apparent from performing Kaplan-Meier survival analysis that the risk of malignant transformation increases as time elapses (see Figure 3).

## 4. Discussion

Primary synovial chondromatosis is a rare disease. Its true incidence is unknown. The incidence of chondrosarcoma in England is however known and is reported to be 1.8/million population per year [15]. Our unit has treated 800 patients with a chondrosarcoma in the same time period as we treated these 78 primary synovial chondromatosis patients. This would suggest that for every 800 chondrosarcomas diagnosed, 5 originated in synovial chondromatosis; that is, 0.6% of chondrosarcomas are linked to primary synovial chondromatosis. As the incidence of chondrosarcoma in England is known, we would expect to see approximately 95 new cases of chondrosarcoma every year. Therefore, every year, 0.57 cases of chondrosarcomas are associated with preexisting synovial chondromatosis; that is, every 1.7 years (20.4 months), 1 new case of chondrosarcoma can be attributed to primary synovial chondromatosis.

Even though our unit is a supra-regional referral unit, it is unlikely that we were referred all patients with synovial chondromatosis over this time period. If we had been then the incidence of primary synovial chondromatosis would be 78/5 of that of chondrosarcoma in primary synovial chondromatosis; that is, 0.06/million per yr, suggesting that there may be 10 new cases of primary synovial chondromatosis diagnosed a year in England. We know no way of confirming this.

Although primary synovial chondromatosis can be locally aggressive with a tendency to recur, it has no metastatic potential. The treatment for symptomatic primary synovial chondromatosis consists of removal of the cartilaginous bodies with or without partial synovectomy. In contrast, chondrosarcoma is a malignant condition requiring more invasive surgery in the form of wide or radical resection or amputation [16] and has a reported incidence of metastases of up to 29% [17, 18].

Clearly because of the differences in prognosis and treatment, it is vitally important to distinguish the benign primary synovial chondromatosis from the malignant chondrosarcoma. In practice, this can be difficult as both conditions have similar patterns of disease presentation with pain, swelling, and restricted joint movement. Numerous authors have found clinical and radiological criteria unhelpful to differentiate between the two disease processes as there is often significant overlap with no definitive features to separate the two [1, 14, 18–20].

Histologically primary synovial chondromatosis reveals lobules of hyaline cartilage that are often hypercellular with atypical histological features, including multinucleation, nuclear crowding, nuclear enlargement, and hyperchromasia with mild myxoid changes often with a variable degree of synovial proliferation or hyperplasia [21]. These atypical features could suggest a malignant neoplasm (grade I or II chondrosarcoma) to the less experienced pathologist when actually they are typical of benign primary synovial chondromatosis [22–24]. Immunohistochemical markers to reliably differentiate between primary synovial chondromatosis and low grade chondrosarcoma would be of great value but, to date, only preliminary studies have been performed [25] with no definitive immunostaining technique being identified as the gold-standard.

The local recurrence of primary synovial chondromatosis has been reported to be as high as 23% [14] despite adequate surgical debridement. The difficulty lies with identifying those recurrences that are benign.

In conclusion, we view multiple recurrences with the development of marrow invasion as highly suspicious of malignant transformation. From our study, it is clear that not all patients with primary synovial chondromatosis require long-term surveillance to monitor for the development of malignant change; however, we recommend that any rapid deterioration in the patient's clinical course, including worsening pain or aggressive recurrence, should be regarded as suspicious and treated at, or referred appropriately to, a tertiary hospital familiar with managing these often complex cases.

## Figures and Tables

**Table 1:** Details of the patients, their tumours, and the outcome.

Case	Age at diagnosis with SC (years)	Site	Treatment(s) for SC	Time to malignant change (years)	Malignancy	Surgical management of chondrosarcoma	Mets	Outcome
1	23	Hip	3 debridements	2.7	Grade I CHS	Hindquarter amputation	—	Alive 13 yrs postoperatively
2	48	Hip	(i) Initially symptomatic (ii) THR 11 yrs after initial diagnosis	11.3	Dedifferentiated chondrosarcoma (osteosarcoma)	Debulking	At presentation (lung)	Died 3 yrs postoperatively
3	25	Hip	(i) 2 arthroscopies (ii) 1 hip resurfacing 17 yrs after diagnosis	20	Grade II CHS	Debulking	At presentation (lung)	Died 4 yrs postoperatively
4	25	Hip	(i) Multiple debridements (ii) THR 2005 (iii) Large recurrence 5 years after THR	39	Grade I CHS	Hindquarter amputation	—	Alive 9 months postoperatively
5	19	Knee	(i) Multiple arthroscopies (ii) TKR aged 55	38.5	Grade II CHS	Above knee amputation	Lung—34 months after amputation	Died 4 yrs postoperatively
